# Tear fluid Calcitonin gene-related peptide in patients with idiopathic intracranial hypertension headache – a prospective case-control study

**DOI:** 10.1186/s10194-026-02453-5

**Published:** 2026-07-24

**Authors:** Cem Thunstedt, Ad Shehi, Ozan E. Eren, Andreas Straube, Katharina Kamm, Ruth Ruscheweyh

**Affiliations:** 1 Department of Neurology, LMU University Hospital, LMU Medizin, Ludwig-Maximilians-Universität München, Munich, Germany; 2 Department of Neurology, Munich Hospital, Bogenhausen, Munich, Germany

**Keywords:** Idiopathic intracranial hypertension (IIH), Pseudotumor cerebri, CGRP, Migraine, Secondary headache, Cerebrospinal fluid

## Abstract

**Background:**

Headache is the most common symptom of idiopathic intracranial hypertension (IIH) and may persist despite therapy, with a significant impact on quality of life. Since calcitonin gene-related peptide (CGRP) plays a crucial role in the pathophysiology and management of primary headaches such as migraine, this raises the question whether CGRP also contributes to headache in IIH. Therefore, we compared tear fluid CGRP levels between IIH patients with headache and healthy controls, and in IIH patients before and after CSF pressure normalization by therapeutic lumbar puncture.

**Methods:**

IIH patients with headache attributed to IIH and healthy controls were included. To avoid confounding with chronic migraine, IIH patients with a chronic migraine phenotype were excluded. Tear fluid was collected from IIH patients and controls. In IIH patients, an additional measurement was performed approximately 3 h after therapeutic lumbar puncture. CGRP levels were analyzed using a commercially available ELISA.

**Results:**

Twenty-three IIH patients (all female; age: 34.0 ± 8.8 years) and 20 healthy controls (all female, age: 25.7 ± 5.5 years) were included. IIH patients had 16.4 ± 12.3 headache days per month and headache was mostly bilateral and pressing. Baseline tear fluid CGRP levels were significantly lower in IIH patients compared to healthy controls (2.4 ± 1.2 ng/ml vs. 4.9 ± 4.2 ng/ml, *p* < 0.001). There was no significant change in CGRP levels in IIH patients before vs. after therapeutic lumbar puncture (2.4 ± 1.2 vs. 2.4 ± 1.7 ng/ml, *p* = 0.236). Similarly, in the subgroup with immediate headache improvement, CGRP levels remained unchanged (before: 2.2 ± 0.9 ng/ml, after: 2.8 ± 2.4 ng/ml; *p* = 0.674).

**Conclusions:**

Tear fluid CGRP levels were lower in IIH patients with headache but without a chronic migraine phenotype compared to healthy controls. In addition, CSF pressure normalization was not associated with changes in CGRP levels after 3 h. These results do not support a major role of CGRP in IIH-associated headache without a chronic migraine phenotype.

**Trial registration:**

The study was previously registered at the German Clinical Trial Register (DRKS www.drks.de) (DRKS00025278), Trial registration date 25.06.2021.

## Background

Idiopathic intracranial hypertension (IIH) is a cerebrospinal fluid (CSF) circulation disorder, predominantly seen in young obese women, leading to elevated intracranial pressure (ICP) without evidence of a structural lesion [1]. The prevalence has been estimated at approximately 9.9 per 100.000 individuals [2].

Patients typically present with chronic headache and progressive visual loss. Hereby, headache is one of the most common symptoms in IIH, typically occurring early in disease course. It often presents with migraine-like quality and moderate to severe intensity, and up to 40% of patients develop a persistent or chronic headache [3, 4]. Fundoscopic examination to detect papilloedema (optic disc swelling) is an important diagnostic step, and MRI imaging of the brain is necessary for differential diagnosis but may also reveal signs of IIH such as (partial) empty sella, posterior globe flattening, cerebral venous sinus stenosis, widening of the perioptic subarachnoid space and increased tortuosity of the optic nerve [5]. However, for confirmation of an IIH diagnosis, observation of the Friedman criteria is required (CSF opening pressure > 25 cmH_2_O, presence of papilloedema or sixth cranial nerve palsy, otherwise normal neurological examination, no structural abnormalities on MRI and normal CSF composition) [6].

Treatment typically includes weight loss in combination with CSF pressure lowering medications such as acetazolamide, topiramate or furosemide. Repeated therapeutic lumbar puncture may be used in selected cases (e.g. vision at risk), but is not recommended as a long-term strategy [7]. In refractory cases, interventions such as sinus stenting, bariatric surgery, fenestration of the optic nerve sheath, and CSF shunting may be considered [7, 8].

Chronic headaches often persist despite adequate CSF pressure control [8] and can substantially impair quality of life [9]. The distinction between IIH-related headache and chronic migraine is challenging, as headache phenotypes frequently overlap and share risk and epidemiological factors as well as overlapping treatment responses (e.g. topiramate) [10].

The neuropeptide calcitonin gene-related peptide (CGRP), which is released by trigeminal nerve endings, is one of the key mediators in migraine pathophysiology [11] and has been clinically established as a target in both acute and preventive migraine treatment [12]. Migraine medications such as monoclonal CGRP antibodies and sumatriptan can also alleviate headache in IIH [13, 14] but confounding with comorbid chronic migraine is difficult to rule out. Therefore, the question arises if CGRP plays a role in IIH-associated headache. One proposed mechanism is that rising intracranial pressure could trigger CGRP-release from trigeminal nerve fibres in dural veins or sinuses, leading to headache [10, 14].

Previous results have been inconclusive. One study showed significantly higher CGRP levels in IIH patients compared to healthy controls [15] while another study found no significant difference [16] and a third study found lower CGRP levels in IIH patients than in controls [17]. Measuring CGRP in tear fluid may provide additional information as it has the advantage of proximity between site of release and site of sampling, reducing dilution, which might lead to a larger sensitivity for detecting group differences [11]. In addition, regarding the mechanisms proposed above, it would be important to know if CGRP levels change after normalization of CSF pressure.

Main objective of our study was therefore to test the hypothesis that tear fluid CGRP is elevated in IIH patients with headache compared to healthy controls, and reduced after normalization of CSF opening pressure. To avoid confounding with chronic migraine, we only included IIH patients without a chronic migraine phenotype.

## Methods

### Participants

This monocentric, prospective case-control study was performed at a tertiary outpatient headache center (Upper Bavarian Headache Center, Department of Neurology, LMU University Hospital Munich). The study was conducted in accordance with the Declaration of Helsinki and was approved by the ethics committee of the medical faculty of the Ludwig-Maximilians-University Munich *(No. 21–0149).* All patients gave their written informed consent.

Patients ≥ 18 years with a diagnosis of idiopathic intracranial hypertension according to Friedman criteria [6] and headache attributed to IIH according to ICHD-3 criteria [18], who were scheduled for a CSF opening pressure measurement as part of their routine treatment at our outpatient clinic and had a CSF opening pressure > 25 cmH_2_O on the experimental day were included. All patients underwent a standardized clinical interview, assessing headache by ICHD-3 criteria and clinical work-up. Neuroimaging showed no parenchymal abnormalities or secondary causes of intracranial hypertension such as venous sinus thrombosis or hydrocephalus. Measurement of CSF opening pressure was performed in lateral decubitus position with legs in a relaxed position, and up to 30 ml of CSF were drained until CSF opening pressure was reduced to the normal range. Exclusion criteria were: any preexisting chronic primary headache disorders (such as chronic migraine or chronic tension type headache) or secondary headache disorders. Preexisting episodic migraine or episodic tension-type headache were allowed. In an attempt to avoid confounding between headache attributed to IIH and chronic migraine, we also excluded all IIH patients exhibiting a chronic migraine phenotype. Other exclusion criteria were wearing contact lenses on the day of tear fluid collection, arterial hypertension (previously diagnosed or values > 140/90 mmHg on the experimental day) due to potential effects on CGRP levels [11], use of CGRP-targeting therapies within the last 3 months or use of acute pain-medication within the last 48 h. In addition, we excluded patients with severe internal, other neurological or psychiatric disorders.

**Fig. 1:**
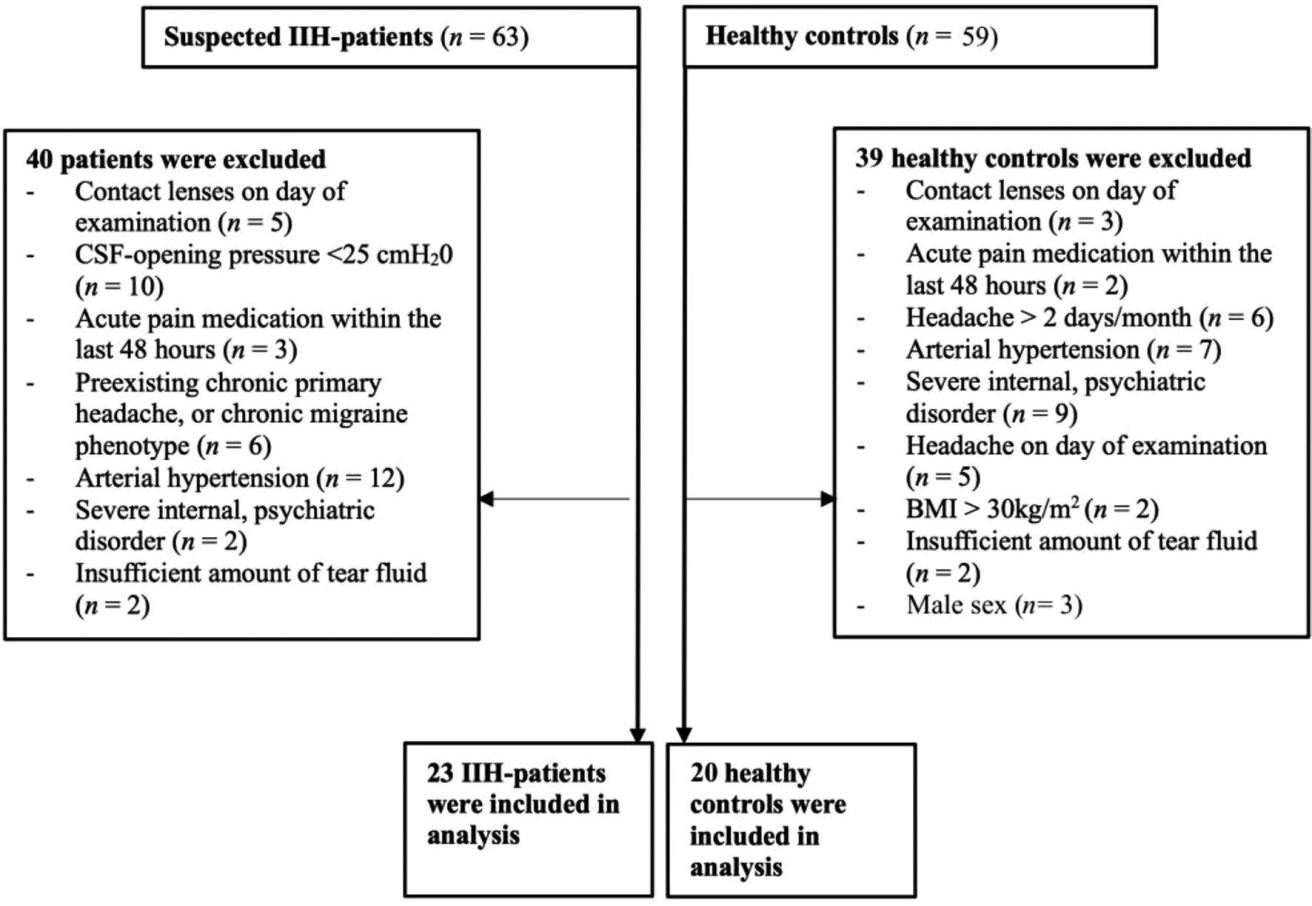
Patient disposition flow chart

**Fig. 2:**
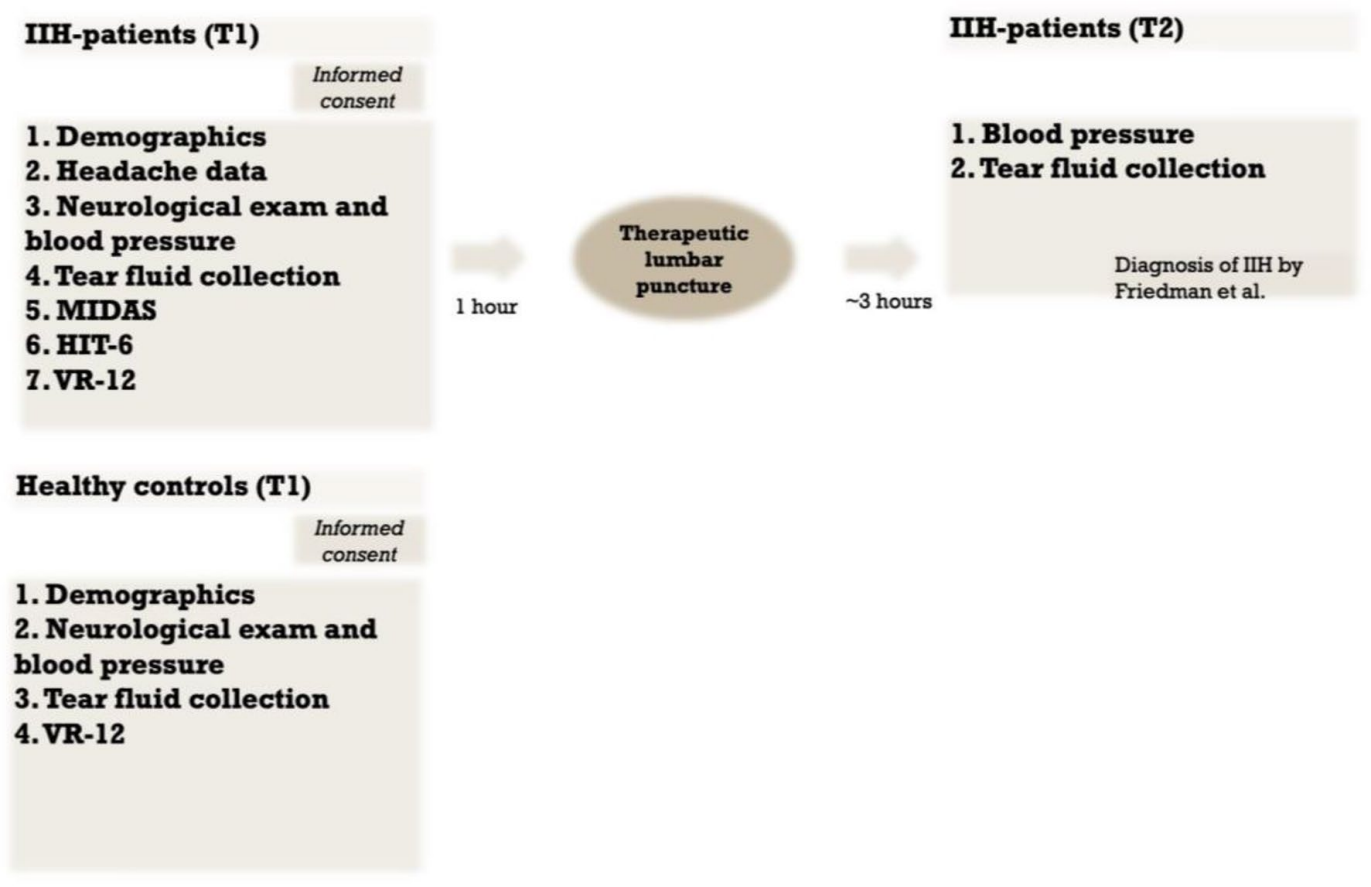
Study workup and timeline. Timeline of the experimental protocol and timing of tear fluid sampling

The control group consisted of healthy volunteers ≥ 18 years with normal neurological examination, recruited on site. Exclusion criteria were: any primary or secondary headache disorder exceeding 2 mild (NRS [0–10] ≤ 3) headache days per month, BMI > 30 kg/m^2^, use of acute pain medication within the last 48 h, severe internal or psychiatric disorder or headache on the experimental day. As the IIH group was purely female, we also excluded male patients. The patient disposition is shown in Fig. 1 and the study procedures in Fig. 2.

### Data collection

The following data were recorded on the experimental day: age, sex, BMI, comorbidities, headache frequency, current and pre-existing headache characteristics (duration, intensity, quality, localization, photo/phonophobia, nausea, dizziness, aura), IIH symptoms (diplopia, blurry vision, scotomas, transient visual obscurations, tinnitus), and medication (acute and preventive headache medication, use of acetazolamide, topiramate or furosemide for IIH treatment, other medication). Several patient-reported outcomes (PROs) were collected in the IIH group: Migraine Disability Assessment (MIDAS) [19], Headache Impact Test (HIT-6) [20], and Veterans Rand 12-Item Health Survey (VR-12) [21]. VR-12 is the license-free alternative to the SF-12, a well-established health-related quality of life measure [21]. VR-12 scores were also obtained in the control group. In IIH patients, we additionally recorded the CSF opening pressure and the drained CSF volume.

### Tear fluid collection

Sampling was conducted as previously described [22]. In IIH patients, the first sample was obtained between 8:00 and 9:00 a.m. (approximately 1 h before lumbar puncture) and the second sample approximately 3 h after therapeutic lumbar puncture. After a 5-minute rest and blood pressure measurement, tear fluid was collected separately from the right and left eye at the lateral canthus using a plastic capillary without irritating the eye (*KABE Laboratories GmbH*,* Nümbrecht*,* Germany*). Tear fluid volume was recorded for each sample and later used to calculate the dilution. The filled capillary was immediately placed into a 1.5 ml tube containing 500 µl T-PER buffer (*Thermo Scientific™*,* Waltham*,* MA*,* USA*), directly placed on ice and centrifuged for 5 min with 4000 rpm at 4 °C. The sample was then stored at -80 °C until analysis.

### CGRP measurement

Tear fluid CGRP concentrations were determined using a human α-CGRP Enzyme-linked Immunosorbent Assay (ELISA) kit (*Abbexa*, *Cambridge*,* UK* [Standard 3.13–200 pg/ml], minimal detectable dose 1.88 pg/ml, intra-array variability < 8%), strictly adhering to manufacturer’s directions. A duplicate standard curve was generated for every batch using a four-parameter logistic regression (*4PL; arigobio.com data calculator*), with all assays achieving a R^2^ > 0.99. Each tear fluid sample was measured in duplicate and results were averaged. Optical densities (OD) were measured with a microplate reader (*PerkinElmer*,* Waltham*,* MA*,* USA*). Mean intra-assay variability was < 8%. Dilution factors were calculated for each sample (500 µl buffer divided by tear fluid volume) to determine final CGRP concentrations.

We chose the Abbexa ELISA because its detection range is suitable for tear fluid samples, that need to be diluted before assessing. The Abbexa kit was developed to detect alpha-CGRP. Recovery after spiking with alpha-CGRP was between 85 and 104%, and recovery after 1:2 to 1:8 dilution was between 80 and 105%, according to (unpublished) information provided by the manufacturer. The Abbexa kit has been validated regarding linearity of different dilutions, and intra- and inter-assay coefficients of variation [23] and has been used in previous CGRP research [24, 25]. Furthermore, it was recently used for CGRP detection in tear fluid by our group, supporting methodological comparability [26].

### Statistical analysis

For statistical analysis SPSS (*SPSS version 29*,* IBM*,* SPSS Inc.*,* Chicago*,* IL*,* USA*) was used. Descriptive statistics are presented as mean ± standard deviation (SD). We conducted non-parametric tests as data were non-normally distributed (according to Shapiro-Wilk test). Nonetheless, data are presented as mean ± SD for comparability with previous studies. Significance was considered at p-value < 0.05 (*two-tailed*). Group differences in age and BMI were analyzed using a Mann-Whitney U test. Categorical variables were compared between groups using the chi-square test (*X*^*2*^), or *Fisher´s exact test*, when expected cell counts were less than 5. Mann-Whitney U test was used for CGRP level comparisons between groups, e.g. between patients and healthy controls. For comparison of CGRP levels before and after therapeutic lumbar puncture, Wilcoxon signed-rank test was applied. Correlations were tested using Spearman correlation.

Primary endpoints were (1) the difference in tear fluid CGRP levels between IIH patients (before lumbar puncture) and healthy controls and (2) the difference in CGRP levels in IIH patients before and after therapeutic lumbar puncture. Exploratory analyses were performed in IIH subgroups, comparing patients with high and low headache frequency, with and without current acetazolamide treatments and with and without immediate headache improvement after therapeutic lumbar puncture.

## Results

We included 23 IIH patients (all female; age 34.0 ± 8.8 years) and 20 healthy controls (all female, age: 25.7 ± 5.5 years). The IIH group was significantly older than the control group (*Z* = − 3.452, *p* < 0.001), The body mass index (BMI) was significantly higher in the IIH group compared with healthy participants (35.2 ± 7.2 kg/m² vs. 24.3 ± 3.3 kg/m²; *Z* = − 4.943, *p* < 0.001).

Regarding quality of life, VR-12 Physical Component Score (PCS) and Mental Component Score (MCS) were significantly lower (indicating lower quality of life) in IIH patients than in controls (IIH: PCS: 37.3 ± 11.2, MCS: 41.6 ± 11.5 vs. controls: PCS: 52.5 ± 4,4; MCS: 54.6 ± 6.0, PCS: *Z* = − 4.7554, *p* < 0.001; MCS: *Z* = − 3.580, *p* < 0.001).

### Headache characteristics

Headache and IIH characteristics are listed in Table 1. In the IIH group, all patients (per inclusion criteria) reported headache, which was mostly bilateral (*n* = 21, 91.3%) and pressing (*n* = 19, 82.6%). Mean monthly headache days (MHD) were 16.4 ± 12.3 and mean monthly acute medication days were 5.2 ± 4.5. Headache intensity on the NRS [0–10] was 6.3 ± 1.5. All IIH patients reported headache on the experimental day. MIDAS scores were 40.2 ± 38.5, and HIT-6 scores were 61.7 ± 10.3, both demonstrating severe headache-related disability on average.

In the control group, 17 participants (85.0%) reported occasional headache, that was (per inclusion criteria) of low frequency (0.9 ± 0.1 days per month) and mild intensity (1.8 ± 0.6 NRS).

**Table 1:** Description of headache and IIH in the IIH group (*n* = 23)

**Description of headache**	
Monthly headache days^a^	16.4 ± 12.3
Monthly acute medication days^a^	5.2 ± 4.5
Headache laterality (*n*, %)^b^	
*Unilateral*	2 (8.7%)
*Bilateral*	21 (91.3%)
Predominant headache quality^b^	
*Burning*	2 (8.7%)
*Pressing*	19 (82.6%)
*Stabbing/Throbbing*	2 (8.7%)
Intensity of headache (NRS [0–10])^a^	6.3 ± 1.5
Nausea or vomiting^b^	4 (17.4%)
Photophobia^b^	1 (4.3%)
Phonophobia^b^	8 (34.8%)
**Description of IIH** ^**a**^	
Time (months) since IIH diagnosis	14.6 ± 13.3
**IIH symptoms** ^**b**^	
Blurred vision	19 (82.6%)
Double vision	9 (39.1%)
Transient visual obscurations (TVO)	11 (47.8%)
Tinnitus	18 (78.3%)
**Abnormal neurological/ophthalmological exam findings** ^**b**^	23 (100.0%)
Sixth cranial nerve palsy	5 (21.7%)
Visual field defect	4 (17.4%)
Papilloedema	19 (82.6%)
**Current acetazolamide use** ^**b, c**^	14 (60.9%)
**Acetazolamide daily dosage (mg/day)** ^**d**^	1000 (500–4000)
**CSF measurements on experimental day** ^**a**^	
CSF opening pressure (cmH_2_O)	32.3 ± 5.5
CSF opening pressure after CSF drainage (cmH_2_O)	18.4 ± 2.7
Amount of CSF drained (ml)	15.0 ± 7.0
Immediate headache improvement after CSF removal in patients^b^	8 (34.8%)
**Patient reported outcomes (PROs)** ^**a**^	
MIDAS score	40.2 ± 38.5
HIT-6 score	61.7 ± 10.3
VR-12 Physical component score	37.3 ± 11.2
VR-12 Mental component score	41.6 ± 11.5

^a^Data are presented as mean ± standard deviation (SD)

^b^Data are presented as absolute frequencies (percentages)

^c^No patient used other IIH medications such as topiramate or furosemide

^d^Data are presented as median and range

Abbreviations: CSF, cerebrospinal fluid; HIT-6, Headache Impact Test-6; IIH, idiopathic intracranial hypertension; MIDAS, Migraine Disability Assessment; NRS, numeric rating scale, PROs, patient-reported outcomes; TVO, transient visual obscurations; VR-12, 12-Item Short Form Veterans Health Survey

### IIH characteristics

See Table 1 for an overview. IIH patients had a history of 14.6 ± 13.3 months since diagnosis and 14 IIH patients were currently using acetazolamide for IIH treatment. No patient used topiramate or furosemide, or any headache/migraine preventive medication. In addition to headache, further current IIH symptoms were blurred vision in 19 patients, double vision in 9 patients and transient visual obscurations in 11 patients. Tinnitus was present in 18 patients. Papilloedema was present in 19 patients at examination. In patients without papilloedema, sixth cranial nerve palsy was present, thereby fulfilling Friedman criteria of IIH [6]. CSF opening pressure on the experimental day was 32.3 ± 5.5 cmH_2_O. No significant correlations were found between CSF opening pressure and BMI (*rho =* 0.103, *p =* 0.641, *n* = 23), or between CSF opening pressure and MHD (*rho =* 0.265, *p =* 0.222, *n* = 23).

### Tear fluid CGRP in IIH patients vs. controls

Tear fluid CGRP levels from the right and left eye were significantly correlated both in IIH patients (before lumbar puncture, *rho* = 0.638, *p* < 0.001, *n* = 23) and in controls (*rho* = 0.774, *p* < 0.001, *n* = 20) Therefore, CGRP levels from the left and right eye were averaged for analysis.

**Fig. 3:**
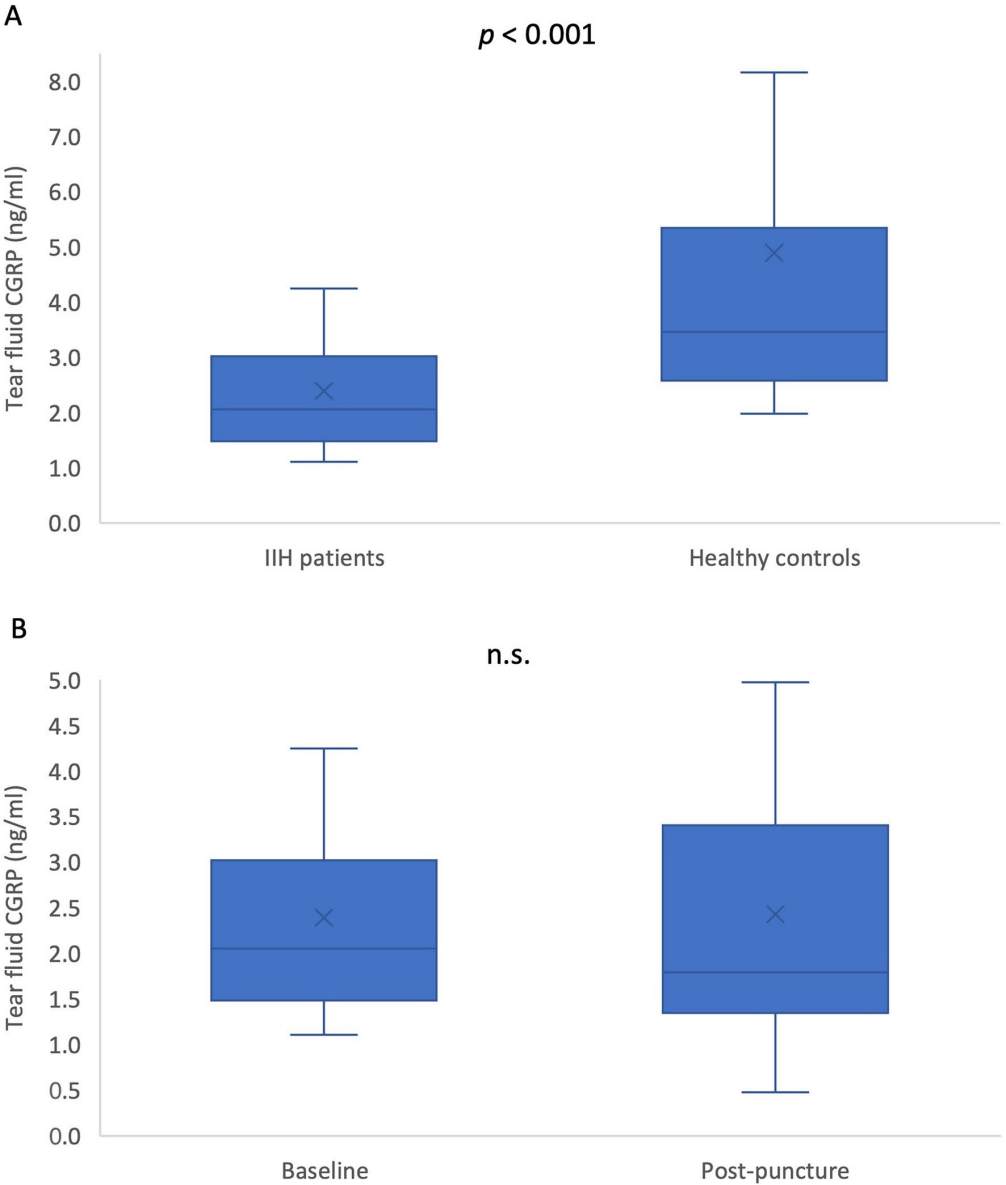
Tear fluid CGRP levels in IIH patients vs. controls and in IIH patients before and after therapeutic lumbar puncture. (**A**) Tear fluid CGRP levels in IIH patients and controls. (**B**) Tear fluid CGRP levels in IIH patients before and after therapeutic lumbar puncture. Boxplots show median and IQR; whiskers indicate range, x indicates the mean. Abbreviations: CGRP, calcitonin gene-related peptide; IIH, idiopathic intracranial hypertension; n.s., not significant

Tear fluid CGRP levels in IIH patients (before lumbar puncture) were significantly lower than in controls (2.4 ± 1.2 ng/ml vs. 4.9 ± 4.2 ng/ml; Z = − 3.385, *p* < 0.001, *n* = 43, Fig. 3).

In IIH patients, there was no correlation of CGRP levels with headache intensity (NRS), neither generally (*rho* = − 0.391, *p* = 0.065, *n* = 23), nor on the experimental day (*rho* = − 0.412, *p* = 0.051, *n* = 23). There was also no correlation of CGRP with MHD (*rho* = − 0.071, *p* = 0.749, *n* = 23) or CSF opening pressure (*rho* = − 0.250, *p* = 0.250, *n* = 23). We also did not find significant correlations between tear fluid CGRP levels and BMI in IIH patients (*rho* = 0.235, *p* = 0.280, *n* = 23) nor in controls (*rho* = 0.033, *p* = 0.890, *n* = 20), or between CGRP and age in IIH patients (*rho* = − 0.275, *p* = 0.203, *n* = 23) or controls (*rho* = - 0.216, *p* = 0.360, *n* = 20).

### Tear fluid CGRP in IIH patients before vs. after therapeutic lumbar puncture

Tear fluid CGRP levels were unchanged 3 h after therapeutic lumbar puncture (before: 2.4 ± 1.2 ng/ml, after: 2.4 ± 1.7 ng/ml, *Z = − 1*.186, *p* = 0.236, *n* = 23, Fig. 3). CGRP levels before and after lumbar puncture were significantly correlated (*rho* = 0.470, *p* = 0.024, *n* = 23).

### Exploratory IIH subgroup analyses

#### Headache frequency

IIH patients with ≥ 15 MHD (*n* = 12) showed somewhat higher tear fluid CGRP levels (before lumbar puncture) compared to patients with < 15 MHD (*n* = 11), but without statistical significance (2.6 ± 1.4 vs. 2.1 ± 0.7 ng/ml, *Z* = − 0.492, *p* = 0.622).

#### Current acetazolamide treatment

No significant differences in tear fluid CGRP levels (before lumbar puncture) were observed between IIH patients with (*n* = 14) and without (*n* = 9) current acetazolamide treatment (2.5 ± 1.5 vs. 2.3 ± 0.9 ng/ml, *Z* = − 0.149, *p* = 0.881).

#### Headache frequency and acetazolamide treatment

There was no significant difference in dosage between patients with ≥ 15 MHD and those with MHD < 15 (1000 ± 790 vs. 1600 ± 1431 mg, *Z* = − 0.914, *p* = 0.361).

#### Immediate headache improvement after therapeutic lumbar puncture

The group of patients with an immediate headache improvement after lumbar puncture (*n* = 8) was analysed separately from the group without immediate improvement (*n* = 15). In the improvement group, CGRP levels did not significantly change after lumbar puncture (before: 2.2 ± 0.9 ng/ml; after: 2.8 ± 2.4 ng/ml; *Z* = − 0.420 *p* = 0.674). Similarly, no significant change was observed in the group without immediate improvement (before: 2.5 ± 1.3 ng/ml, after: 2.2 ± 1.3 ng/ml; *Z* = − 1.874, *p* = 0.061). The mean change in CGRP levels from before to after lumbar puncture was 0.6 ± 2.6 ng/ml in the improvement group and – 0.3 ± 0.6 ng/ml in the group without improvement (*Z* = − 0.968, *p* = 0.333).

### Discussion

The main result of our study was that tear fluid CGRP levels in IIH patients were significantly lower than in healthy controls. In addition, CGRP levels did not change within 3 h after normalization of intracranial pressure by therapeutic lumbar puncture. All patients suffered from IIH with headache, but patients with a chronic migraine phenotype were excluded. Although part of the patients already received IIH treatment, all still had increased CSF opening pressure on the experimental day.

In our sample, tear fluid CGRP levels were lower in IIH patients compared to healthy controls. This is contrary to our hypothesis that CGRP has a role in IIH headache pathophysiology manifesting with increased CGRP levels. However, also previous studies have found numerically or significantly lower CGRP values in IIH patients compared to healthy controls [16, 17]. The study by Krajnc et al. found significantly lower plasma CGRP in 26 IIH patients compared to 57 healthy controls [17]. Within the IIH patients, those with a migraine-like phenotype had higher CGRP than those with non-migraine headache, and those without headache had lowest CGRP levels. However, even in the migraine-like phenotype, levels were still lower than in healthy controls [17]. In the study by Hansen et al., plasma CGRP levels were numerically (but not significantly) lower in 97 newly diagnosed IIH patients compared to 37 healthy controls [16]. No significant differences were found between IIH patients with and without headache. There were also no group differences in CSF CGRP levels. This is the largest study to date, and has the advantage to be matched for BMI. However, the study did also not find the expected differences between healthy controls and chronic migraine, and it has been discussed if sample processing and storing may have compromised CGRP detection [16]. Finally, one study reported higher plasma CGRP levels in IIH with migraine-like headache compared with healthy controls [15]. In this study, plasma CGRP was compared between patients with IIH, episodic and chronic migraine, and healthy controls showing that CGRP levels in IIH were significantly higher than in healthy controls but significantly lower than in chronic migraine. BMI was similar between groups [15]. It has been discussed if lower CGRP levels in IIH patients in the Krajnc et al. study may be due to BMI differences between groups [17]. In our study, only participants with BMI < 30 kg/m^2^ were allowed into the control group, because we thought that obese controls might have a risk of undiscovered intracranial hypertension. This naturally resulted in a group difference in BMI. However, there was no correlation between CGRP and BMI, neither in the IIH group nor in the control group. Similarly, the Hansen et al. study did also not find a correlation between plasma CGRP levels and BMI [16], and an older study on obesity even found higher plasma CGRP levels in obese women compared to normal weight controls [27]. Moreover, tear fluid CGRP should be less sensitive to the effects of higher blood volume and consecutive larger dilution than serum or plasma CGRP. In summary, we believe that BMI differences cannot explain our results of lower CGRP values in the IIH group. Another factor that could influence results is the headache phenotype. The Krajnc et al. study found higher CGRP levels in IIH patients with migraine-like headaches than on those with non-migraine headaches [17]. In our study, we excluded IIH patients with a chronic migraine phenotype to avoid confounding with chronic migraine, which is known to be associated with increased CGRP levels [11, 28, 29]. This might have contributed to lower CGRP levels in our IIH group. Notably, the only study finding increased CGRP levels in IIH patients compared to healthy controls [15] included only IIH patients with migraine-like headache. Although this is purely speculative, our and previous data might be consistent with IIH itself being associated with lowered CGRP values that would be masked by a concurrent migraine-like headache associated with increased CGRP levels. The question remains why CGRP levels should be reduced in IIH patients with non-migraine headache. It has been proposed that adaptive changes e.g. desensitization related to the chronic headache course could contribute to reduced CGRP levels [30]. However, this does not seem to be the case in chronic migraine patients, that have elevated CGRP values [11, 28, 29].

We did not find any changes in tear fluid CGRP within 3 h after CSF pressure normalization by therapeutic lumbar puncture, neither in the total group nor in the group reporting immediate headache relief after lumbar puncture. One might argue that the 3-hour window is too short to expect a change, however, fast changes in CGRP levels occur, e.g. after intake of an acute headache medication [26, 31]. Nonetheless, it would have been preferable to have an additional data point e.g. after 24 h, but this was not acceptable to patients in our outpatient setting where many patients cover long distances to come to our clinic. However, our results are consistent with the Krajnc et al. study, which observed patients for 6 months of treatment with a total of 8 datapoints, and did not find longitudinal changes in CGRP levels [17]. Also, the Ak et al. study found no change of CGRP levels from the baseline to the control visit after treatment, independent of the headache course [15]. It has been discussed if CGRP is related to other types of pain, although the evidence is not as clear as for migraine [32]. Therefore, it cannot be excluded that CGRP levels increased by procedural pain might have confounded a CGRP reduction by intracranial pressure lowering and subsequent headache reduction. This could only be amended by comparing patients undergoing lumbar puncture with and without CSF drainage.

Although CGRP levels were somewhat higher in IIH patients with higher headache frequency in our study, this did not reach significance. Given the limited sample size, these results should be interpreted with caution. However, Kranjc et al. also did not find a correlation between CGRP levels and headache frequency in IIH patients [17].

In summary, previous and present results suggest that CGRP may not play the main pathophysiological mechanism in IIH headache. Possible non-CGRP mechanisms of IIH-related headache could include direct irritation of trigeminal receptors or neuroplastic changes. Additionally, other neuropeptides (like PACAP) or metabolic dysregulations associated with IIH, such as insulin resistance, might contribute to IIH and headache by stimulating CSF production and leading to a pro-inflammatory state that increases trigeminal sensitivity [33, 34].

Thus, available data suggest that headache in IIH patients may comprise more than one component. While a purely pressure-related headache might be independent of CGRP-mediated mechanisms, a subset of patients appears to suffer from an additional migraine-like headache component, which might exhibit a CGRP pathophysiology. These two entities may share overlapping risk factors (e.g. obesity, metabolic changes) but might have different neurobiological bases. This hypothesis would also explain why migraine-like headaches in IIH are often not satisfactory relieved by intracranial pressure reduction alone, yet may respond to treatment with monoclonal CGRP antibodies, independently of reduction of intracranial pressure or papilloedema [13, 35, 36].

Regarding demographic data, our findings are in line with previous studies. Characteristics of the IIH patients (age, female predominance, and BMI) were comparable [16, 17]. The lack of correlation between BMI and opening pressure is consistent with previously reported heterogenous data on this relationship [37, 38]. Furthermore, there was no significant association between CSF opening pressure and headache frequency, also aligning with previous observations [39].

## Strength and limitations

It is a strength of our study to have used CGRP detection in tear fluid, which has the advantage of proximity between site of release (trigeminal nerve endings) and site of sampling, thereby reducing dilution. It also is a strength that we excluded IIH patients with a chronic migraine phenotype, as this reduces confounding between chronic migraine and IIH related headache. Of course, it would have been even better to investigate two groups of IIH patients, those with and those without a chronic migraine phenotype, and this approach is also considered for future studies in our group. Results would also have been strengthened by comparison with a group of chronic migraine patients to confirm elevated tear fluid CGRP levels in these patients within the same study. Several additional limitations need to be considered. Our study population was small; therefore, subgroup analyses remain purely exploratory. However, main results (difference between IIH patients and controls and lack of change after lumbar puncture) were very clear. Another limitation was the assessment of headache by structured retrospective interview rather than based on detailed headache diary. In addition, our cohorts were (on purpose) not matched for BMI, as discussed above. They were also not perfectly matched for age; however, age was not correlated to CGRP levels, neither in our study nor in previous studies [23]. Also, our previous study found lower tear fluid CGRP levels in healthy controls [26]. This might be due to different factors of inter-study variability of CGRP measurements [23, 25], including longer storage time in the previous study. Nonetheless, within-study comparisons of groups sampled and analysed at the same time, as done in the present study, remain valid analyses.

It must be mentioned that IIH patients were investigated not at the time of their first diagnosis, but mostly after several months of follow-up. Our cohort therefore consisted of a convenience sample of patients still having active IIH (fulfilling Friedman criteria) in spite of several months of treatment according to current management recommendations [7]. Usually, treatment was started with a weight loss intervention plus acetazolamide, and switched to topiramate and/or furosemide if necessary. GLP-1 receptor agonists were not used as they do not have insurance coverage for weight loss treatment in Germany, and their cost mostly precludes patients paying for them out of their own pocket. Our cohort consisted of patients who could not tolerate drug or weight loss treatment, did not sufficiently respond to treatment, or relapsed after initially successful treatment, but papilloedema and/or visual loss were not severe enough to warrant invasive treatment. In this cohort, CSF opening pressure measurement and drainage were typically performed because of persisting or exacerbating visual disturbances, headache, or (mostly mild) papilloedema, relapse with weight gain, as a control after discontinuation of pharmacological treatment, and in patients with IIH without papilloedema to assess response to treatment.

## Conclusion

This is the first investigation of tear fluid CGRP in IIH patients compared to controls, and after therapeutic lumbar puncture. Our study included only IIH patients with headache but without a chronic migraine phenotype. We found lower CGRP levels in IIH patients compared to healthy controls, without significant change after ICP normalization. Our results do not support a major role of CGRP in IIH-associated headache without a chronic migraine phenotype. However, further investigation is needed, preferably including also IIH patients with a chronic migraine phenotype and patients with chronic migraine without IIH, to better disentangle the complex relationship between CGRP, IIH-related headache and migraine.

## Data Availability

The data that support the findings of this study are not openly available due to reasons of sensitivity and are available from the corresponding author upon reasonable request. Data are located in controlled access data storage at LMU University Hospital Munich.

## Abbreviations

BMI: Body Mass Index
CSF: Cerebrospinal Fluid
CGRP: Calcitonin Gene-Related Peptide
DRKS: German Clinical Trial Register
ELISA: Enzyme-linked Immunosorbent Assay
HIT-6: Headache Impact Test
ICHD: International Classification of Headache Disorders
ICP: Intracranial Pressure
IIH: Idiopathic Intracranial Hypertension
SD: Standard Deviation
TPER: Tissue Protein Extraction Reagent
NRS: Numeric Rating Scale
MCS: Mental Component Score
MHD: Monthly Headache Days
MIDAS: Migraine Disability Assessment
MRI: Magnetic Resonance Imaging
OD: Optical Density
PCS: Physical Component Score
PRO: Patient-Reported Outcomes
VR-12: Veterans RAND 12-Item Health Survey
